# Plemorphic Adenoma of the Infratemporal Space: A New Case Report

**DOI:** 10.1155/2009/529350

**Published:** 2010-02-07

**Authors:** Tarik El-Hadi, A. Oujilal, M. Boulaich, L. Sqalli, M. Kzadri

**Affiliations:** Service ORL, Hôpital des Spécialités, Rabat-Instituts, Rabat 6220, Morocco

## Abstract

Plemorphic adenoma is a frequent benign tumor of the major salivary glands. It could also develop from accessory salivary glands. We are reporting an extremely rare case of pleomorphic adenoma developing from the infratemporal space. The final diagnosis was based on histological confirmation. The treatment was mainly a complete resection via an anterior transmaxillary approach. Diagnosis, clinical behaviour, and treatment of pleomorphic adenoma of the infra-temporal space are reviewed from the literature.

## 1. Introduction

Salivary gland tumors represent 3% of all head and neck tumors. Plemorphic adenomas originate from the salivary glands, with a rate of 85 to 90% in the major salivary glands and 6% in the minor salivary glands mainly the oral cavity, especially the palatal mucosa [1]. 

Scarcely, pleomorphic adenoma is found in the nasal cavity, pharynx, larynx, trachea, and lacrimal glands [2].

 Plemorphic adenoma of the infratemporal space is extremely rare, and to our knowledge, only two cases have been reported in the English literature [3, 4].

## 2. Case Report

A 59-year-old woman presented with a 6-month history of painless and gradually increasing cheek swelling. Extra-oral examination showed a right painless and firm subzygomato-malar mass. Intraoral examination revealed a firm, nodular swelling obliterating the right part of the superior buccal vestibule. The overlying gingival mucosa was normal. Oropharynx and swallowing were intact. Nasal airflow, anterior rhinoscopy, and nasofibroscopy were strictly normal. Examination of the major salivary glands was unremarkable. No cervical lymph nodes were found, and the remainder of the physical examination was within normal limits. 

A computed tomography scan without administration of IV contrast revealed a moderate sized and well-limited isodense ovalar lesion (33 × 18 mm) in the right infratemporal space, indenting upon the posterolateral wall of the right maxillary antrum. There was no destruction of adjacent bone structures. Lateral nasal wall, nasal turbinates, nasopharynx, orbits, parotid glands, submandibular glands, and sinuses were all normal (Figure 2). MRI was not performed because of patient's financial constraints. 

Neither incisional biopsy nor fine needle aspiration was performed for the simple reason that we believed they increase the risk of recurrence and cause local spread of the tumor. After careful review of the literature, this attitude seems to be totally wrong. In fact, FNA and incisional biopsy must be performed in every case of parapharyngeal and infratemporal space tumor. 

A surgical resection was decided via the transmaxillary approach through a 6 cm right superior vestibular incision from tooth 12 to tooth 16. Adequate access to the tumor was obtained, after the resection of anterior and medial walls of the maxillary sinus, exposing thereafter its posterior wall which was pushed forward by the tumor, allowing a complete en bloc resection of the mass without any injury to adjacent structures in the infratemporal space. 

The excised tumor was sent for histopathological examination. On macroscopic examination, the tumor mass was whitish, lobulated, well limited, and encapsulated, weighing 17 g and measuring 4 × 3.5 × 2 cm in size.

In paraffin sections, histological features of the tumor included a mixture of epithelial, myoepithelial, and myxochondroid stroma. 

**Figure 1:**
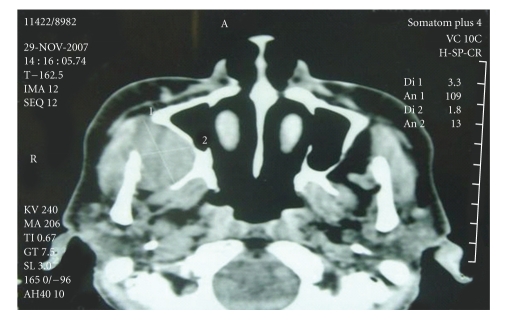
CT scan showing a well limited solid tumor developing from the right infratemporal space indenting upon the posterolateral wall of the right maxillary sinus.

**Figure 2:**
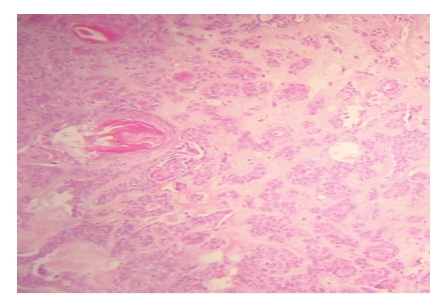
Histologic findings of the tumor showing the ductal epithelial and myoepithelial elements with chondromyxoid stroma (×8; H&E).

The histopathological findings were consistent with a pleomorphic adenoma (Figure 1).

The patient remained free of disease 12 months after surgery. 

## 3. Discussion

Pleomorphic adenoma or mixed tumor is the most common benign tumor arising from both the major and minor salivary glands.

Approximately 90% of pleomorphic adenomas occur in the major salivary glands, and 6% in the minor salivary glands [1]. They rarely occur at other sites in the upper aerodigestive tract including the nasal cavity, pharynx, larynx, trachea, and lacrimal glands [2].

Unique cases have been reported in the literature regarding head and neck cutaneous locations of pleomorphic adenoma, including the scalp, eyelids, nose, cheek, upper lip, external ear, and external auditory canal [5].

It is necessary to point out, at this level, that this case report is about a pleomorphic adenoma arising from the infratemporal space, which has been reported in the English literature only twice [3, 4].

The first case was published in 2000 in the European archives of otorhinolaryngology about a 52-year-old woman with a swelling in the left buccal area. CT scan showed a localised moderate sized tumor in the left pterygopalatine fossa. The surgical resection was performed via the transmaxillary approach. The second case published in 2007 in Head and Neck Pathology was about a 45-year-old woman with a right cheek swelling and a nodular swelling filling the superior ipsilateral oral vestibule. CT scan revealed a moderate sized lesion in the right retromaxillary space with anterior bowing of the posterolateral wall of the maxillary sinus. Surgical resection of the tumor was performed through the transzygomatic approach. Both histopathologic evaluations were compatible with a typical pleomorphic adenoma.

Multiple hypotheses have been raised regarding the origin of all these abnormal locations of salivary gland tissue. Ferlito has already noticed the existence of heterotopic salivary gland tissue in head and neck region, especially in the pituitary gland, external auditory canal, nasal fossae, sterno-clavicular joint, mandibula, and cervical soft tissues. All these heterotopic salivary gland tissues have a potential development of pleomorphic adenoma [6].

Generally, benign tumors of the infratemporal space remain asymptomatic for a long time.

They are usually revealed by a swelling on the face, with Intraoral expression of the mass: nodular swelling obliterating the superior buccal vestibule. 

 Endoscopic examination of the nasal cavities is essential in the sense that it may reveal any involvement of the lateral nasal wall which could be pushed back by the tumor. 

CT scan is a crucial tool which has been proven to be of significant utility in determining the exact volume and location of the tumor, its extensions and its connections with neighboring structures. Contrast enhancement is mainly found in vascular and neurogenic tumors. If a tumor is doubtful to be vascular, MRI angiography or conventional arteriography must be performed.

Only when the vascular nature of the tumor is ruled out, fine needle aspiration or incisional biopsy can be performed prior to surgery, in order to get the primary histological diagnostic.

Pleomorphic adenoma is histologically characterized by a mixture of epithelial, myoepithelial, and stromal elements. The stroma may be myxoid, chondroid, or hyaline.

The treatment of pleomorphic adenoma of the infratemporal space is exclusively surgical. 

Many surgical approaches have been described in literature. The transmaxillary approach is opted for when the tumor has a moderate volume and a limited local propagation. 

 This approach avoids the risk of damage to the facial nerve and is preferable cosmetically.

The lateral facial approach is indicated when the mass is enormous and very extensive. It carries the risk of facial nerve injury and may leave a disgracious scar.

While many authors described that complete surgical resection is the only treatment for pleomorphic adenoma, some reports showed good results with adjuvant radiotherapy against inoperable tumors after incomplete resection [7]. However it still remains controversial.

In spite of its benign character, pleomorphic adenoma remains a tumor which has a very high potential of local recurrence ranging from 2.4 to 10% [8], a potential of malignant transformation varying from 6 to 10% [9], and a risk of distant metastasis [10]. 

Long-term follow-up is therefore mandatory, even if the tumor is completely resected and appears to be clinically and histologically benign.

## 4. Conclusion

Pleomorphic adenoma in the infratemporal space is very rare. Clinical examination is usually poor and CT scan is considered to be the key investigation. MRI is indicated when a vascular tumor is suspected. Incisional biopsy guides the diagnostic. Surgical resection is the main treatment and the transmaxillary approach is preferred when the tumor has a moderate volume and a limited local extension. The high potential of recurrence of pleomorphic adenoma makes the follow-up mandatory over many years after surgery.
